# Leaf extract of *Caesalpinia mimosoides* enhances oxidative stress resistance and prolongs lifespan in *Caenorhabditis elegans*

**DOI:** 10.1186/s12906-019-2578-5

**Published:** 2019-07-08

**Authors:** Panthakarn Rangsinth, Anchalee Prasansuklab, Chatrawee Duangjan, Xiaojie Gu, Krai Meemon, Michael Wink, Tewin Tencomnao

**Affiliations:** 1 Graduate Program in Clinical Biochemistry and Molecular Medicine, Department of Clinical Chemistry, Faculty of Allied Health Sciences, Chulalongkorn University, Bangkok, 10330 Thailand; 2 College of Public Health Sciences, Chulalongkorn University, Bangkok, 10330 Thailand; 3 Department of Biotechnology, School of Environmental and Chemical Engineering, Dalian Jiaotong University, Dalian, 116028 China; 4 Department of Anatomy, Faculty of Science, Mahidol University, Bangkok, 10400 Thailand; 5 Institute of Pharmacy and Molecular Biotechnology, Heidelberg University, Im Neuenheimer Feld 364, 69120 Heidelberg, Germany; 6 Age-Related Inflammation and Degeneration Research Unit, Department of Clinical Chemistry, Faculty of Allied Health Sciences, Chulalongkorn University, Bangkok, 10330 Thailand

**Keywords:** *Caesalpinia mimosoides*, *Caenorhabditis elegans*, Antioxidant, Oxidative stress, Aging, DAF-16

## Abstract

**Background:**

*Caesalpinia mimosoides*, a vegetable consumed in Thailand, has been reported to exhibit in vitro antioxidant properties. The in vivo antioxidant and anti-aging activities have not been investigated. The aim of this research was to study the antioxidant activity of *C. mimosoides* extracts in *Caenorhabditis elegans,* a widely used model organism in this context.

**Methods:**

*C. elegans* were treated with *C. mimosoides* extracts in a various concentrations. To investigate the protective effects of the extract against oxidative stress, wild-type N2 were used to determine survival rate under oxidative stress and intracellular ROS. To study underlying mechanisms, the mutant strains with GFP reporter gene including TJ356, CF1553, EU1 and LD4 were used to study DAF-16, SOD-3, SKN-1 and GST-4 gene, respectively. Lifespan and aging pigment of the worms were also investigated.

**Results:**

A leaf extract of *C. mimosoides* improved resistance to oxidative stress and reduced intracellular ROS accumulation in nematodes. The antioxidant effects were mediated through the DAF-16/FOXO pathway and SOD-3 expression, whereas the expression of SKN-1 and GST-4 were not altered. The extract also prolonged lifespan and decreased aging pigments, while the body length and brood size of the worms were not affected by the extract, indicating low toxicity and excluding dietary restriction.

**Conclusions:**

The results of this study establish the antioxidant activity of *C. mimosoides* extract in vivo and suggest its potential as a dietary supplement and alternative medicine to defend against oxidative stress and aging, which should be investigated in intervention studies.

**Electronic supplementary material:**

The online version of this article (10.1186/s12906-019-2578-5) contains supplementary material, which is available to authorized users.

## Background

It is well established that the consumption of vegetables and fruits is important for human health. Attention has been focused on the effects of bioactive secondary metabolites of plant foods on the prevention of diseases related to oxidative stress. Many secondary metabolites, such as polyphenols, ascorbic acid and carotenoids, can reduce the oxidative stress generated by reactive oxygen species (ROS) [1, 2].

ROS are a free radicals that play a role in aging and oxidative stress-related conditions [3]. The production of ROS is a consequence of both exogenous and endogenous factors. Exogenous factors include pollution, ultraviolet radiation and/or unhealthy life habits such as smoking and a diet low in antioxidants [4]. Endogenous factors are related to cellular metabolism, where most of the ROS production occurs during mitochondrial respiration. The antioxidant defense system coevolved along with aerobic metabolism to counteract damage from ROS [5]. When the generation of ROS is not in balance with antioxidant activity, ROS can damage biomolecules, including lipids, proteins and DNA. These activities can mediate aging as well as other chronic diseases such as atherosclerosis, cardiovascular diseases, myocardial infarction, stroke, chronic inflammation, rheumatoid arthritis, cancer, diabetes, septic shock, and other degenerative diseases in humans [3, 6, 7].

*Caesalpinia mimosoides* Lam. [recently, reclassified as *Hultholia mimosoides* (Lam.) E. Gagnon & G. P. Lewis], a native plant of the northern and northeastern parts of Thailand that is locally called Pak Nam Puya in Thai, also occurs in other areas of tropical eastern Asia. This legume (Fabaceae, subfamily Caesalpinioideae) is a small spiny tropical tree or climbing shrub. Leaves and young twigs of the plant are edible. They are usually consumed in Thailand as a fresh vegetable side dish or appetizer [8]. In addition, the plant has been traditionally used as an anti-flatulent and a remedy against fainting and dizziness. [8] A leaf extract of *C. mimosoides* has also been found to exhibit antioxidant [8], anti-inflammatory [9], anticancer [10] and antimicrobial activities [11]. *C. mimosoides* has been reported to contain multiple phenolic compounds, including gallic acid and flavonoids which are known antioxidants [8, 12]. However, in vivo studies of its antioxidant and anti-aging properties have not yet been reported.

*Caenorhabditis elegans*, a free-living soil nematode, is considered the simplest major multicellular model organism for studying genetic and pharmacological influences on aging and longevity because of its short lifespan, similarities with the human aging process and susceptibility to oxidative stress [13]. Moreover, the genome of *C. elegans* has been completely sequenced. It carries many homologous genes implicated in human diseases. Major signaling pathways that regulate longevity and stress resistance are well conserved in *C. elegans* [14–16]. Recent reports suggest that plant extracts with high concentrations of phenolic secondary metabolites exhibit antioxidant and anti-aging activities in *C. elegans* [17–21].

In this present study, leaves and young twigs of *C. mimosoides* (CM) were exhaustively extracted with hexane, ethyl acetate, and methanol in a Soxhlet apparatus. We selected the methanol extract of CM which exhibited highest polyphenol and flavonoid contents for subsequent experiments. We used this extract to examine in vivo antioxidant and lifespan extension activities in *C. elegans.* Furthermore, the extracts were also investigated to rule out toxicity and a potential influence of dietary restriction.

## Methods

### Chemicals

Dimethyl sulfoxide (DMSO), 2,2′-azino-bis (3-ethylbenzothiazoline-6-sulfonic acid) diammonium salt (ABTS), 2,2-diphenyl-1-picrylhydrazyl (DPPH), Folin-Ciocalteu reagent, quercetin, 5′-fluorodeoxyuridine (FUdR), ampicillin and epigallocatechin gallate (EGCG) were purchased from Sigma-Aldrich (St. Louis, MO, USA). Gallic acid was purchased from TCI America (Portland, OR, USA), L-ascorbic acid from Calbiochem (San Diego, CA, USA), 2,7-dichlorofluorescein diacetate (H_2_DCF-DA) from Fluka Chemie GmbH (Buchs, Switzerland), juglone (5-hydroxy-1,4-naphthalenedione) from Sigma-Aldrich GmbH (Steinheim, Germany), and sodium azide from AppliChem GmbH (Darmstadt, Germany). Other reagents used in the extraction process were of analytical grade and purchased from RCI Labscan (Bangkok, Thailand).

### Plant material and extraction

In this study, we used leaves and young twigs, approximately 15–20 cm in length from the top of the tree shoots of *C. mimosoides* (CM) collected from the local market in Chiang Rai Province, Thailand. The plant was authenticated and identified with voucher specimen number A014170 (BCU) at the herbarium of Kasin Suvatabhandhu (Department of Botany, Faculty of Science, Chulalongkorn University, Thailand).

Plant material was extracted by the Soxhlet procedure. Briefly, the young twigs and leaves were dried in a ventilated incubator at 40 °C and ground into a fine powder. Then, approximately 40 g of the dried powder was uniformly packed into a thimble and sequentially extracted in a Soxhlet apparatus with 400 mL of three different extracting solvents (hexane, ethyl acetate, and methanol) for at least 24 h per solvent. The resulting supernatants were collected, filtrated and evaporated to dryness under vacuum. The yields of hexane, ethyl acetate and methanol extraction were 4.02, 6.29 and 29.82% (w/w), respectively. Finally, each extract was prepared as a stock solution of 100 mg/mL in DMSO, sterilized through a 0.2 μm pore size syringe filter, stored at − 20 °C, and protected from light until further use.

### Quantification of Total phenolic content

The total phenolic content was determined by the Folin-Ciocalteu method. The assay was modified for a microplate format as described previously [21–23]. Briefly, 50 μL of the extract (1 mg/mL) was mixed thoroughly with 50 μL of a 10-fold diluted Folin-Ciocalteu reagent. After 20 min incubation, the mixture was neutralized by addition of 50 μL of a 7.5% (w/v) Na_2_CO_3_ solution and then kept in the dark at RT for a further 20 min. Finally, the absorbance was measured at 760 nm using a microplate reader (BioTek Instruments, Winooski, VT, USA). The content of total phenolics was calculated from a standard calibration curve of gallic acid and the results were expressed as mg of gallic acid equivalent (GAE) per g of dry weight extract.

### Quantification of flavonoid content

The total flavonoid content was determined by an aluminum chloride (AlCl_3_) colorimetric method based on the formation of yellow colored aluminum-flavonoid complexes under alkaline condition. The assay was modified for a microplate format, as previously described [21–23]. In brief, 50 μL of the extract (1 mg/mL) was made up to 200 μL with 95% ethanol, and mixed well with 10 μL of 10% (v/v) AlCl_3_ solution and 10 μL of 1 M sodium acetate (NaOAc) solution. Then the mixture was allowed to stand for 40 min in the dark and the absorbance was measured at 415 nm using a microplate reader (BioTek Instruments, Winooski, VT, USA). The content of total flavonoids was calculated from a standard calibration curve of quercetin and the results were expressed as mg of quercetin equivalent (QE) per g of dry weight extract.

### DPPH assay

The 2,2′-diphenyl-1-picrylhydrazyl (DPPH) assay was used to evaluate the free radical scavenging activity of the extract based on its hydrogen atom- or electron-donating capacity to neutralize the stable radical DPPH (DPPH•), accompanied by a color change from purple to yellow. The assay was performed by using a microplate format. For the assay protocol, 100 μl DPPH• working solution (0.2 mM) was added to 100 μl of the extract at a ratio of 1:1 (v/v). The reaction mixture was incubated in the dark at RT for 30 min, and the absorbance was recorded in a microplate reader (BioTek Instruments, Winooski, VT, USA) at 517 nm. Radical scavenging activity was expressed as the percent inhibition of the DPPH• radicals calculated by the following equation: % Inhibition = 100 x [Abs of control - (Abs of sample - Abs of blank) / Abs of control]. Ascorbic acid (vitamin C) and EGCG were used as controls to study the effective concentration (EC_50_) of the extract.

### ABTS assay

The 2,2′-azinobis-(3-ethylbenzothiazoline-6-sulfonic acid) (ABTS) assay was used to evaluate the free radical scavenging activity of the extract based on its hydrogen atom- or electron-donating capacity to neutralize the stable free radical cation ABTS (ABTS•+), accompanied by a color change from green to colorless. The cation radical ABTS• + working solution was generated by the oxidation of 7 mM ABTS with 2.45 mM potassium persulfate (K_2_S_2_O_8_) at a 1:1 (v/v) ratio. The assay was performed by using a microplate format, in which the reaction consisted of 100 μl ABTS• + working solution and 100 μl extracts at a 1:1 (v/v) ratio. The mixture was then incubated in the dark at RT for 45 min, and the absorbance was recorded using a microplate reader (BioTek Instruments) at 734 nm. Radical scavenging activity was expressed as the percent inhibition of the ABTS• + radicals calculated by the following equation: % Inhibition = 100 x [Abs of control - (Abs of sample - Abs of blank) / Abs of control]. Ascorbic acid (vitamin C) and EGCG were used as controls to study the effective concentration (EC_50_) of the extract.

### Qualitative phytochemical profiling - LC-MS

The methanol extract of *C. mimosoides* was submitted to the Institute of Systems Biology (Universiti Kebangsaan Malaysia, Malaysia) to screen for phytochemicals with liquid chromatography-mass spectrometry (LC-MS) analysis. The analytical system used was a DionexTM UltiMate 3000 UHPLC system (Thermo Fisher Scientific) coupled with a high-resolution micrOTOF-Q III (Bruker Daltonik GmbH, Bremen, Germany). The chromatographic separation was performed on an AcclaimTM Polar Advantage II C18 column (3 mm × 150 mm, 3 μm particle size) (Thermo Fisher Scientific) with a gradient mobile phase consisting of 0.1% formic acid in water (solvent A) and 100% acetonitrile (solvent B). The elution program was as follows: 5% B (0–3 min); 80% B (3–10 min); 80% B (10–15 min) and 5% B (15–22 min). The flow rate was 400 μL/min within a 22 min total run time, and the injection volume was 1 μL. The MS instrument was operated in the positive electrospray ionization (ESI) mode with the following parameters: drying gas flow at 8 L/min; drying gas temperature at 200 °C; nebulizer pressure at 1.2 bar; capillary voltage at 4500 V; and m/z scan range of 50 to 1000. For identification of putative compounds, the observed (experimental) m/z values were compared with the METLIN (CA, USA) and the KNApSAcK (Keyword Search Web Version 1.000.01) databases as well as with the calculated (theoretical) mass values from available previously published data, with an accepted difference of less than 30 ppm (ppm). The relative abundance of a compound is expressed as the percentage of the peak area relative to the total area of all peaks observed in the chromatogram.

### *Caenorhabditis elegans* strains, maintenance, synchronization and treatment

The *C. elegans* strains used in this study were wild type N2, BA17 (fem-1(hc17)IV), TJ375 (gpIs1[hsp-16.2::GFP]), TJ356 (zIs356[daf-16p::daf-16a/b::GFP + rol-6]), CF1553 (mu1s84[pAD76(sod-3::GFP)]), LD1 (ldIs7 [skn-1b/c::GFP + rol-6(su1006)]), CL2166 (dvIs19[pAF15(gst-4::GFP::NLS)]), and CF1038 (daf-16(mu86)I). The worms were all cultured with nematode growth medium (NGM) containing *E. coli* OP50 as a food source and kept in a 20 °C incubator. All strains and *E. coli* OP50 were obtained from Caenorhabditis Genetics Center (CGC), University of Minnesota, USA.

Age synchronization of the worms was achieved by isolating eggs from gravid hermaphrodites. The eggs were prepared by adding lysis solution containing 5 M NaOH and 5% NaOCl, followed by vortexing for 10 min and centrifuging for 2 min at 1800 rpm. Then, the supernatant was removed, and the pellet was washed once in sterile water before centrifugation for an additional 2 min. After discarding water, the remaining eggs were resuspended in M9 buffer for hatching. Larvae were then kept after hatching in S-medium containing *E. coli* OP50 (OD_600_ = 1.0). Different treatments were applied according to each experiment. Agar diffusion test was performed to exclude the antimicrobial activity of CM extract against *E. coli* OP50 (see Additional file 1).

For the experiment, the worms were divided into four groups. The first group was treated with 1% DMSO (solvent control group). This group served to exclude any toxicity of the solvent used for dissolving the extracts on worms. Groups two through four were treated with 25, 50 and 100 μg/ml CM extracts dissolved in DMSO (maximum 1%), respectively.

### Survival assay under Juglone-induced oxidative stress

The assay was modified as previously described [19, 24]. Age synchronized L1 larvae stage of wildtype N2 and transgenic CF1038 (DAF-16 loss-of-function mutant) worms were divided into four groups of 80 worms each and treated with different concentrations of CM extracts or with DMSO diluted in S-medium and bacteria, as mentioned above. After 48 h of treatment at 20 °C, the pro-oxidant juglone (a naphthoquinone from *Juglans regia*) was added to a final concentration of 80 μM, which is a lethal concentration, prior to incubation at 20 °C for an additional 24 h. Afterwards, surviving and dead worms were counted.

### Intracellular ROS accumulation

Age-synchronized N2 and CF1038 worms (L1 stage) were treated with CM extracts or DMSO in S-medium at 20 °C for 48 h. Each group contained 200–300 individuals. Then, 50 μM H_2_DCF-DA was added and incubated for 1 h away from light at 20 °C. After that, the worms were mounted on a glass slide and paralyzed by the addition of 10 mM sodium azide, and at least 30 worms were randomly photographed using a fluorescence microscope BIOREVO BZ-9000 with a mercury lamp (Keyence Deutschland GmbH, Neu-Isenburg, Germany) with λex 480/20 nm, λem 510/38 nm, 10X objective lens and constant exposure time. ImageJ software version 1.50i (National Institutes of Health, Bethesda, MD, USA) was then used to measure the relative fluorescence intensity of the full body [19, 24].

### HSP-16.2 expression

L1 age-synchronized TJ375 transgenic worms, which express a HSP-16.2::GFP reporter gene, were treated with CM extracts or DMSO as previously mentioned and incubated at 20 °C for 72 h. Then, the nematodes were exposed to a nonlethal dose of 20 μM juglone for 24 h. The worms were then mounted on a glass slide with 10 mM sodium azide, and images of at least thirty worms per group were taken with a 20X objective lens at constant exposure time via fluorescence microscopy. Analysis of at least three replicates was performed by quantifying the mean relative fluorescence intensity of the pharynx using ImageJ software [24].

### Subcellular DAF-16 localization

TJ356 transgenic L1 worms, which express DAF-16::GFP fusion protein, were treated with CM extracts or DMSO as previously described and kept at 20 °C. After 72 h, the worms were mounted on a glass slide using 10 mM sodium azide. At least thirty worms per group were imaged on a fluorescence microscope with a 10X objective lens and constant exposure time. Distribution of the transcription factor DAF-16::GFP in each worm can be in the nucleus, cytoplasm, or the intermediate region between the nucleus and cytoplasm. Worms were sorted and counted according to localization of DAF-16::GFP [19].

### Subcellular SKN-1 localization

LD-1 transgenic worms, which express a GFP reporter-fused SKN-1, were age-synchronized at the L1 stage and were treated with CM extracts or DMSO as described previously and kept at 20 °C for 48 h. Fluorescence intensity was measured by fluorescence microscopy as described above. Then, the worms were mounted on a glass slide using 10 mM sodium azide for paralysis, and at least thirty worms per group were visualized under a fluorescence microscope at a 20X objective lens and constant exposure time. The transcription factor SKN-1::GFP in each worm can be located in the nucleus, cytoplasm or the intermediate between the nucleus and cytoplasm. The nematodes were sorted and counted according to the SKN-1::GFP subcellular localization.

### SOD-3 expression

Age-synchronized CF1553 transgenic worms, expressing SOD-3::GFP fusion protein, at the L1 stage were treated with CM extracts or DMSO as described above and cultured at 20 °C for 72 h. After treatment, the worms were mounted on a glass slide with 10 mM sodium azide, and at least thirty worms per group were imaged using a fluorescence microscope with a 10X objective lens and constant exposure time. The experiment was repeated at least three times, and analysis was performed by measuring the relative fluorescence intensity using ImageJ software [19, 24].

### GST-4 expression

At the L1 stage, synchronized CL2166 worms expressing GST-4::GFP fusion protein were treated with CM extracts or DMSO as described above and kept at 20 °C for 48 h. After treatment, the worms were exposed to 20 μM juglone and incubated at 20 °C for 24 h. Then, the worms were paralyzed with 10 mM sodium azide on a glass slide, and at least thirty worms were imaged using a fluorescence microscope with a 10X objective lens at constant exposure time. Analysis of three replicates was performed by measuring the relative fluorescence intensity using ImageJ software.

### Lipofuscin level

BA17 transgenic worms, which are thermosensitive and do not lay eggs at 25 °C, were used to measure the expression of lipofuscin, an autofluorescent pigment that accumulates over time and thus is an indicator of aging. The worms at the L1 larval stage were treated with CM extracts or DMSO as mentioned above and cultured at 25 °C for 16 days. The media and treatments were changed every second day. At day 16, the worms were paralyzed with 10 mM sodium azide on a glass slide, and at least thirty randomly selected worms were imaged on a fluorescence microscope at a 10X objective lens and constant exposure time. Three repeat experiments were performed by measuring the relative fluorescence intensity using ImageJ software.

### Longevity assay

N2 synchronized L4 larvae were plated on NGM agar plates containing a lawn of ampicillin-resistant *E. coli* OP50 supplemented with CM extract at a concentration that exhibited the best antioxidant capacity in the worms. The NGM plates also contained 50 μM of 5′-fluorodeoxyuridine (FUdR) to prevent the growth of progeny and 0.1 mg/mL of ampicillin (Amp) to prevent foreign bacterial contamination. The worms were grown at 25 °C, examined under a stereomicroscope (Nikon Corporation, Tokyo, Japan) and counted daily starting from the first day (day 0) that they were transferred to experimental NGM plates until all individuals had died. The worms were scored as dead when they no longer responded to gentle stimulus with a platinum wire and showed no pharyngeal pumping movement. Worms with internally hatched progeny or extruded gonads were censored and excluded from the experiment. The experiment was performed with at least 100 worms per group.

### Body length and brood size

N2 worms were age synchronized by picking adult worms into NGM agar plates with *E. coli* OP50 as a food source. The adult worms were allowed to lay eggs for 2–4 h before removal, and then the remaining eggs were incubated at 20 °C for 48 h. After incubation, worms at the L4 larval stage were sorted and used in the experiments. For the body length assay, 50 worms at the L4 larval stage were placed on NGM agar plates supplemented with CM extracts or DMSO in the *E. coli* OP50 lawn as a food source and cultured at 20 °C for 24 h. Adult day 1 worms were paralyzed by using 10 mM sodium azide and mounted on a glass slide. At least thirty worms per group were imaged using a 10X objective lens of bright-field microscope. The software BZ-II Analyzer (Keyence Corp.) was used for the analysis of the body length.

For the brood size assay, each L4 larval stage worm was individually sorted, transferred onto different NGM plates and treated with CM extracts or DMSO. The worms were allowed to grow and lay eggs at 20 °C and were observed under a dissecting microscope. The eggs were counted and removed to separate them from adult worms every day until the adult worm stopped laying eggs.

### Statistical analyses

All experiments were performed in at least triplicate. Total phenolic and total flavonoid content as well as DPPH and ABTS results are presented as the mean ± standard deviation (SD). The data from *C. elegans* experiments are presented as the mean ± standard error of the mean (SEM). The differences between groups were analyzed using one-way analysis of variance (ANOVA) followed by Bonferroni’s method (post hoc). For the lifespan assay, the statistical significance among different groups was determined by a log-rank (Mantel − Cox) test followed by the Gehan-Breslow-Wilcoxon test. Differences with *p* < 0.05 were considered statistically significant.

## Results

### Chemical characterization

**Fig. 1:**
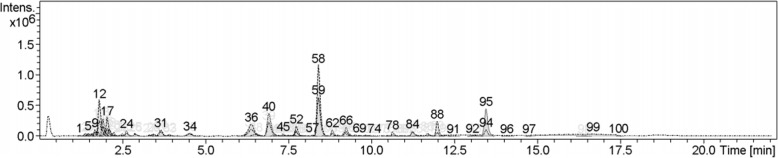
LC-MS profile of CM methanol extract. The total ion chromatogram (TIC) was generated by LC-MS under positive electrospray ionization. Peak numbers correspond to those in Table 1

The methanol extract of CM was investigated by LC-MS. More than hundred peaks were detected (Fig. 1). Chromatographic peaks were tentatively identified by comparing the MS data with databases based on the search of m/z values of molecular ion peaks in the positive mode [M + H]^+^. The five major phytochemical compounds were 3-O-methylgallate, 4-aminomethylindole, emmotin A, theogallin and gallic acid (Table 1).

**Table 1:** Proposed phytochemical constituents in methanol extract of *C. mimosoides* using LC-MS

Peak No.	Rt (min)	[M + H]^+^ (m/z)	Area (%)	Proposed compound	Theoretical mass	Mass error (ppm)
12	1.8	146.0824	5.9	4-Aminomethylindole	145.0766	9
17	2.0	256.1181	3.0	N-D-Glucosylarylamine	255.1107	0
36	6.4	171.0288	4.4	Gallic acid	170.0215	0
40	6.9	345.0818	5.6	Theogallin	344.0743	0
52	7.7	329.0869	1.6	Bergenin	328.0794	0
58	8.4	185.0461	13.0	3-O-Methylgallate	184.0372	8
59	8.4	185.0445	7.2	3-O-Methylgallate	184.0372	0
62	8.8	481.0979	1.1	Quercetin-3′-glucuronide	480.0904	0
66	9.2	465.1032	1.6	Quercetin-3-O-glucoside	464.0955	0
88	12.0	415.2117	2.3	Clausarinol	414.2042	0
95	13.4	279.1588	5.7	Emmotin A	278.1518	1

Database: METLIN (CA, USA) and KNApSAcK Keyword Search Web Version 1.000.01

The observed m/z values of putative compounds were compared with the METLIN (CA, USA) and the KNApSAcK (Keyword Search Web Version 1.000.01) database). The five major phytochemical compounds tentatively identified were 3-O-methylgallate, 4-aminomethylindole, emmotin A, theogallin and gallic acid

### Total phenolic and flavonoid contents

The total phenolic and flavonoid contents in extracts from young twig and leaves of *C. mimosoides* (CM) were determined. The highest total phenolic (460.25 ± 3.08 mg GAE/g dry weight extract) and total flavonoid (12.55 ± 0.43 mg QE/g dry weight extract) contents were found in the methanol extract, followed by the ethyl acetate extract and then the hexane extract (Table 2). Due to these promising results, the CM methanol extract was therefore selected for subsequent experiments.

**Table 2:** Total phenolic and flavonoid contents of *C. mimosoides* extracts

Extraction solvents	Total Phenolic Content	Total Flavonoid Content
	mg GAE/g dry weight extract	mg QE/g dry weight extract
Hexane	5.35 ± 0.85	1.76 ± 0.32
Ethyl acetate	323.21 ± 6.45	8.89 ± 0.39
Methanol	460.25 ± 3.08	12.55 ± 0.43

Values are expressed as the mean ± SD (*n* = 3)

Methanolic extract of CM expressed the highest total phenolic and flavonoid contents when compared to CM extracted by ethyl acetate and hexane

### In vitro evaluation of antioxidant properties

DPPH and ABTS assays were used to investigate the free radical scavenging capacities of CM extracts in vitro. Methanol extracts possessed strong antioxidant activities because they exhibited high scavenging activities against DPPH and ABTS radicals. The effective concentration (EG50) was recorded (Table 3).

**Table 3:** Free radical scavenging capacities of *C. mimosoides* extracts

Samples	EC_50_	
	DPPH assay	ABTS assay
CM methanol (μg/ml)	8.20 ± 0.29	5.16 ± 0.98
Vitamin C (μM)	40.50 ± 0.27	26.99 ± 0.41
EGCG (μM)	15.56 ± 0.10	8.95 ± 0.34

Values are expressed as the mean ± SD (*n* = 3)

CM methanol extract showed antioxidant properties. 8.20 ± 0.29 and 5.16 ± 0.98 μg/ml of the extract were the EC_50_ to scavenge free radical by DPPH and ABTS assay, respectively

### Effect of CM extract against juglone-induced oxidative stress in wild type worms

**Fig. 2:**
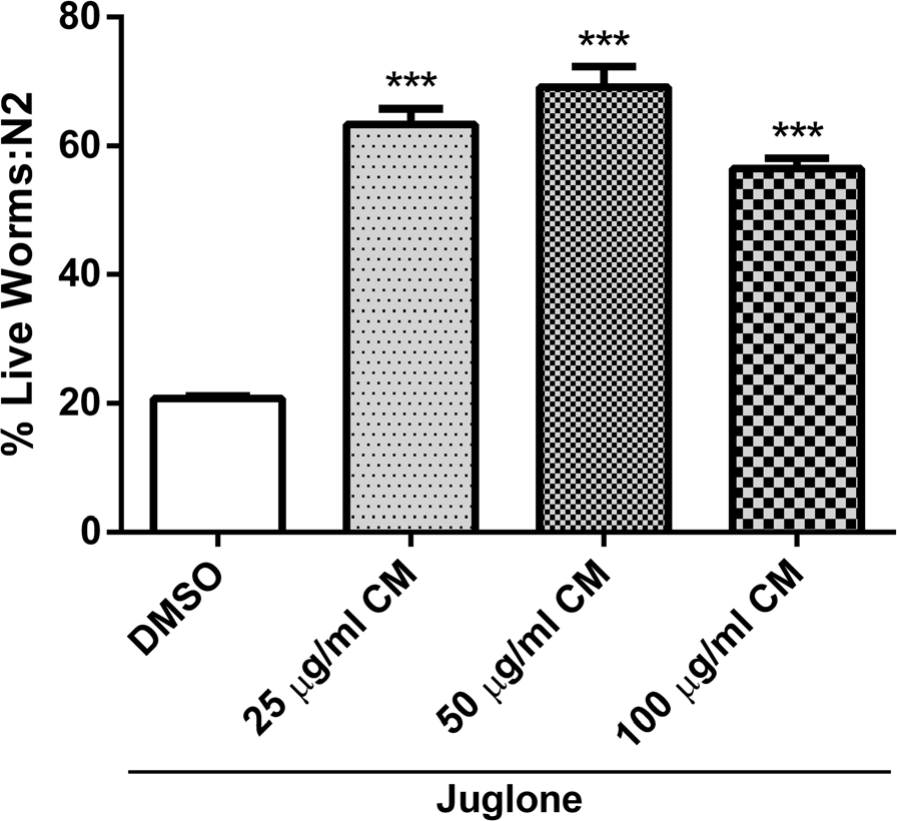
Effect of Different concentrations of CM extract on survival rate under juglone-induced oxidative stress in N2 worms. Survival rate was significantly Increase after CM treatment. Values are mean ± SEM of at least 3 independent experiments. ***Different of DMSO control (*p* < 0.001)

Juglone, a yellow pigmented pro-oxidant from *Juglans regia,* is commonly used to induce ROS-related mortality of *C. elegans* [25]. Only 20% of the worms survived after being treated with 80 μM juglone. However, pretreatment of the worms with methanol extracts (25, 50 and 100 μg/ml) significantly enhanced the survival rates. Among the tested concentrations, 50 μg/ml extract showed the highest survival percentage (69.11 ± 2.36%) when compared to the DMSO solvent control (20.79 ± 0.38%) (*p* < 0.001) (Fig. 2).

### Effect of CM extract on intracellular ROS accumulation in wild type worms

**Fig. 3:**
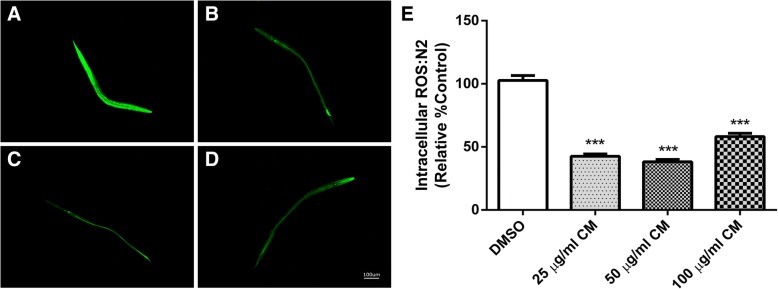
Effect of CM extracts on intracellular ROS accumulation. N2 worms were treated with DMSO (**a**) and CM extracts 25, 50 and 100 μg/ml (**b**-**d**, respectively) incubated with H2DCF-DA for measurement of intracellular ROS accumulation. Intracellular ROS was significantly decrease after CM treatment (**e**). Values are mean ± SEM of at least 3 independent experiments. ***Different of DMSO control (*p* < 0.001)

To further test the antioxidant effect of the methanol extract in vivo, intracellular ROS levels were evaluated in wild type N2 worms using H_2_DCF-DA, a widely known fluorescence probe for detecting intracellular ROS production. The ROS level correlates with the fluorescence intensity resulting from oxidation by ROS, leading to the formation of the highly fluorescent 2′7’-dichlorofluorescin [26]. The results showed a significant decrease in the fluorescence intensity of the extract-treated groups (25, 50 and 100 μg/ml), and the lowest levels were in the group treated with 50 μg/ml extract compared to the DMSO control group (*p* < 0.001) (Fig. 3). 100 μg/ml showed some pro-oxidant activity.

### Effect of CM extract on HSP-16.2 expression

**Fig. 4:**
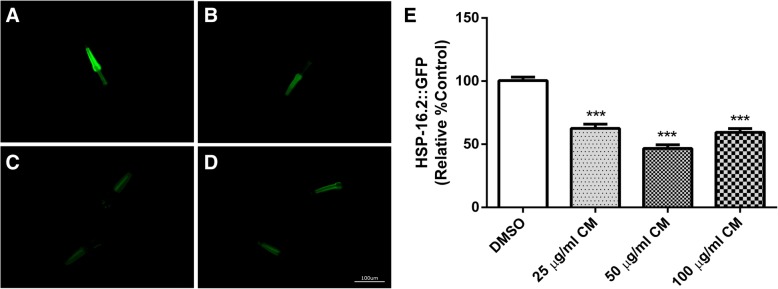
To assessment of HSP-16::GFP expression after CM treatment. TJ375 worms were treated with DMSO (**a**) and CM extracts 25, 50 and 100 μg/ml (**b**-**d**, respectively). The expression of HSP::GFP significantly decrease after CM treatment (**e**). Values are mean ± SEM of at least 3 independent experiments. ***Different of DMSO control (*p* < 0.001)

TJ375 mutant worms express heat shock protein (HSP)-16.2, which is commonly used as a marker of oxidative stress in the nematode [27, 28]. To further test the antioxidant effect of the methanol extract, HSP-16.2 levels were investigated in the worms treated with juglone and the extract. The expression of HSP-16.2 was significantly reduced in worms that were pretreated with the extract (25, 50 and 100 μg/ml) compared to the expression in worms treated with 20 μM juglone alone. Among the concentrations tested, the 50 μg/ml extract treatment showed the lowest expression of HSP-16.2 (46.63 ± 5.93%) when compared to the DMSO control group (*p* < 0.001) (Fig. 4).

### Effect of CM extract on DAF-16/FOXO pathway

**Fig. 5:**
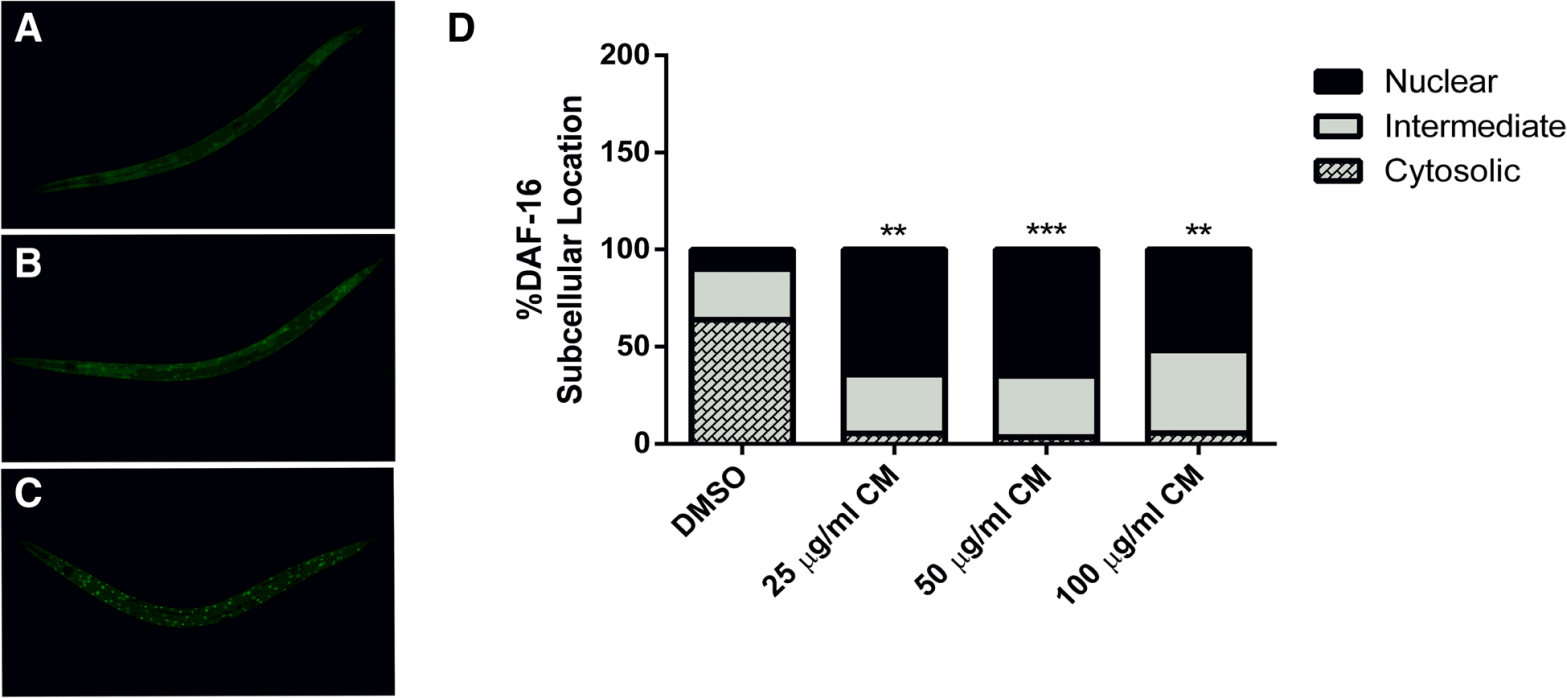
Effect of CM extract on DAF-16 translocation. DAF-16 locations in TJ356 worms: Cytosol (**a**), intermediate (**b**) and nucleus (**c**). CM extract-treated worms significantly increase DAF-16 translocation to the nucleus (**d**). Values are mean ± SEM of at least 3 independent experiments. **Different of DMSO control (*p* < 0.01), ***Different of DMSO control (*p* < 0.001)

To investigate the mechanisms involved in the antioxidant effect of CM, the expression of transcription factor DAF-16, the *C. elegans* homologue to the fork head transcription factor (FOXO) in humans, was examined in TJ356 transgenic worms. Treatment with the extract (25, 50 and 100 μg/ml) enhanced DAF-16 translocation from the cytoplasm to the nucleus. The 50 μg/ml extract resulted in a high percentage of nuclear subcellular localization of DAF-16::GFP (65.02 ± 4.55%) compared to the DMSO control group (9.96 ± 2.97%) (*p* < 0.001). This result suggests that the antioxidant effect of the extract was mediated through the DAF-16/FOXO pathway (Fig. 5).

**Fig. 6:**
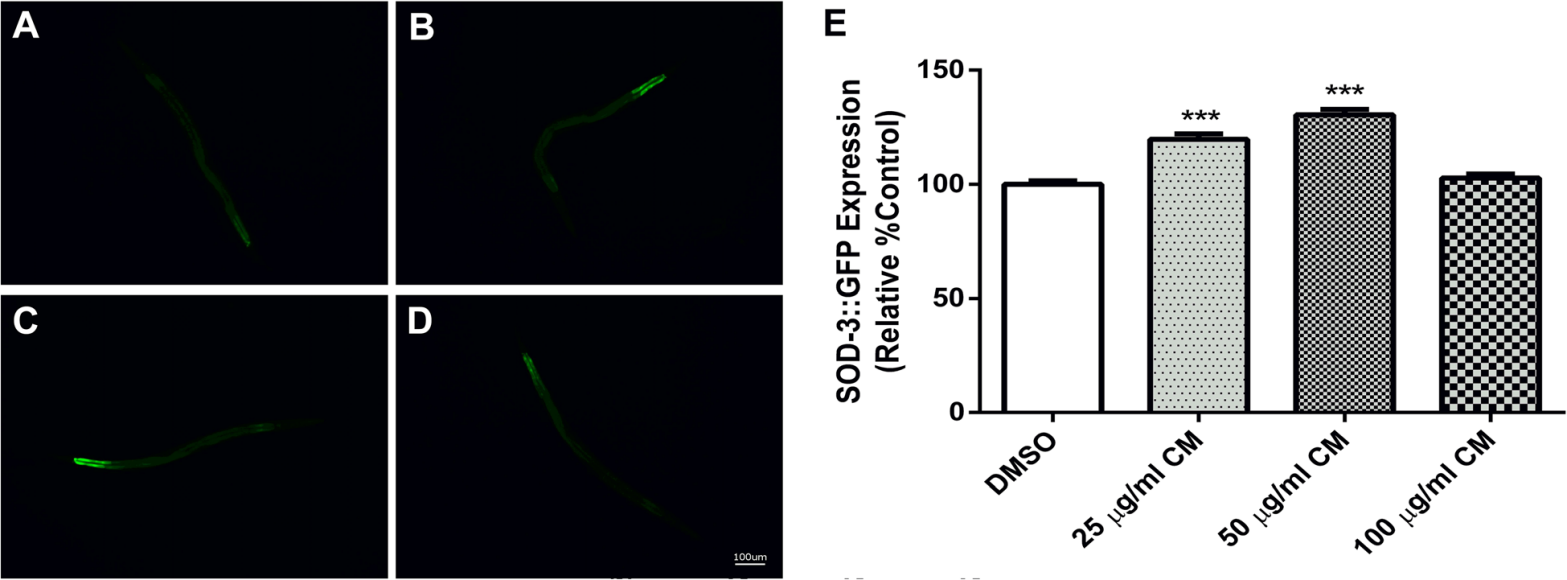
Effect of CM extracts on SOD-3::GFP expression. CF1553 worms were treated with DMSO (**a**) and CM extracts 25, 50 and 100 μg/ml (**b**-**d**, respectively). The expression of SOD-3::GFP significantly enhance after CM treatment (**e**). Values are mean ± SEM of at least 3 independent experiments. ***Different of DMSO control (*p* < 0.001)

Normally, DAF-16 activation subsequently results in the activation of other stress response genes, such as SOD-3, which is a key enzyme that protects the worms against ROS [29]. We found that SOD-3:GFP showed higher expression levels in CF1553 transgenic worms treated with 25 and 50 μg/ml extract (119.8 ± 2.315 and 130.5 ± 2.392, respectively) compared to the DMSO control group (*p* < 0.001). However, 100 μg/ml of the extract did not show any difference in fluorescence intensity compared to the control (Fig. 6).

**Fig. 7:**
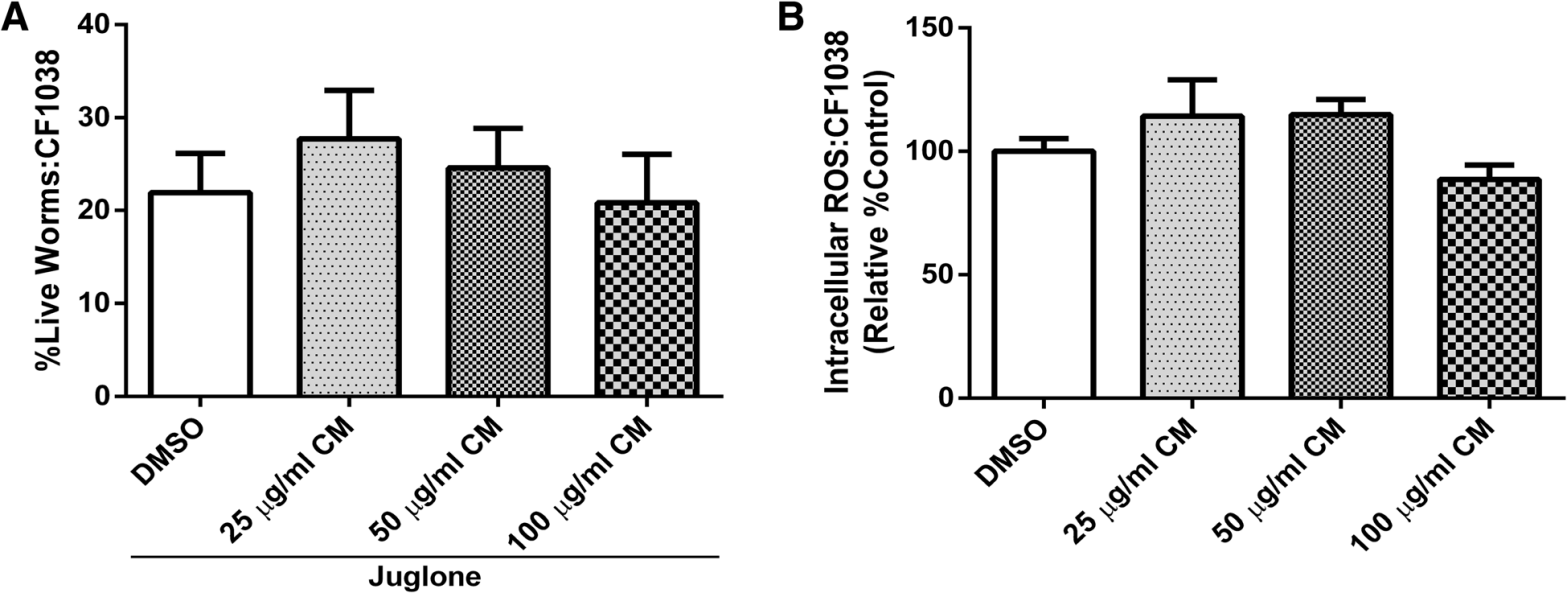
CF1038 (DAF-16 loss of function) worms were used to confirm the effect of CM extract on DAF-16/FOXO pathway by survival assay under juglone-induced oxidative stress (**a**) and Intracellular ROS measurement (**b**) which the worms failed to counteract with stress and reduce intracellular ROS, respectively

To further confirm the antioxidant effect of CM via the DAF-16/FOXO pathway, DAF-16 loss-of-function transgenic worms CF1038 were employed to examine their survival and intracellular ROS levels under juglone-induced oxidative stress. Extract-treated DAF-16 mutant worms were unable to compensate for the mortality under juglone-induced oxidative stress. Likewise, no difference of intracellular ROS accumulation between CM extracts and DMSO control groups was detected (Fig. 7).

### Effect of CM extract on SKN-1/NRF-2 pathway

**Fig. 8:**
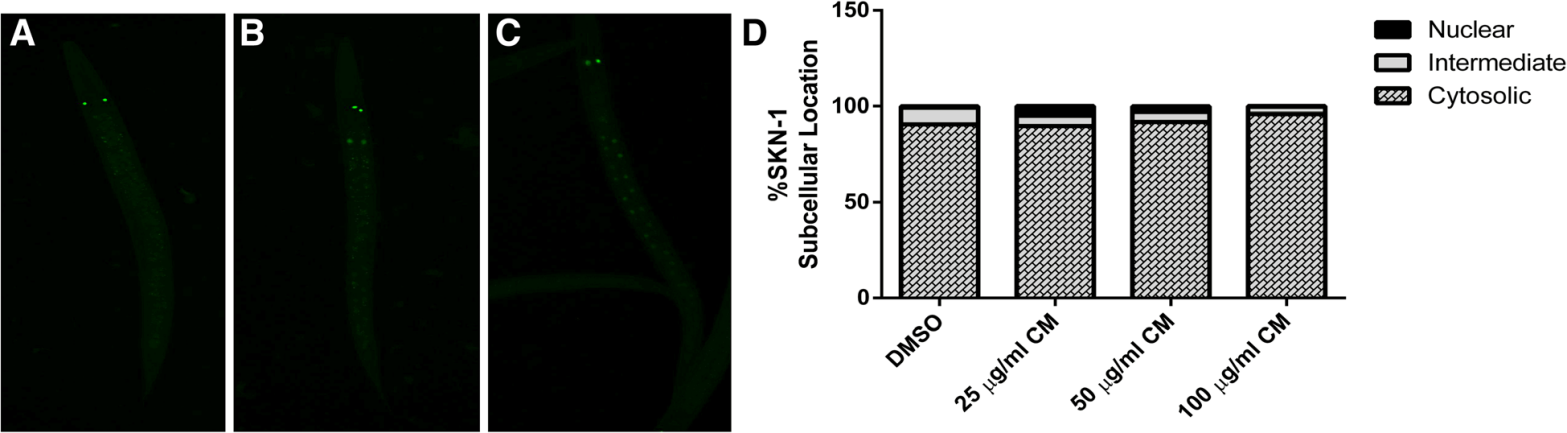
Effect of SKN-1 translocation after treatment with CM extracts. SKN-1 locations in LD1 worms: Cytosol (**a**), intermediate (**b**) and nucleus (**c**). No alteration of SKN-1 subcellular localization was observed (**d**). Values are mean ± SEM of at least 3 independent experiments

SKN-1, the *C. elegans* homologue to the mammalian NRF2 transcription factors, is known as a major regulator of antioxidant response in *C. elegans*. LD-1 transgenic worms, treated with 25, 50 and 100 μg/ml extracts did not show any difference in SKN-1 nuclear translocation when compared to DMSO control group (Fig. 8).

**Fig. 9:**
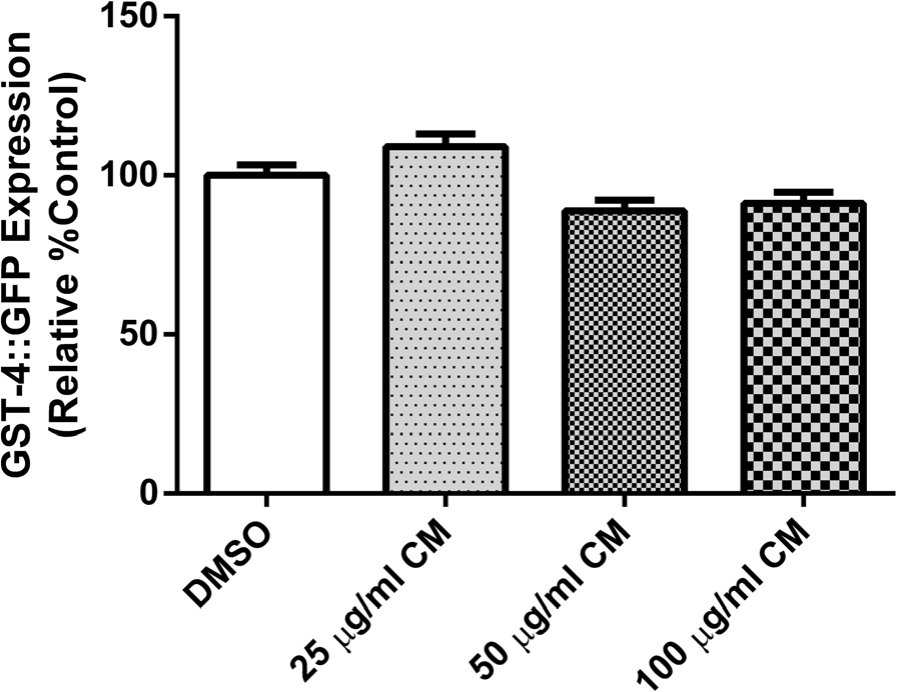
Effect of CM extracts on GST-4::GFP expression. CL2166 worms were treated with DMSO and CM extracts 25, 50 and 100 μg/ml for measurement of GST-4::GFP expression (E). Values are mean ± SEM of at least 3 independent experiments

GST-4 is an isoform of the glutathione S-transferases, which are involved in the worm’s response to oxidative stress. This gene is regulated by the SKN-1 transcription factor. These results support the SKN-1 experiments (Fig. 8) in that extracts (25, 50 and 100 μg/ml) had no effect on the expression of GST-4 in CL2166 worms when compared to the DMSO control (Fig. 9).

### Effect of CM extract on lipofuscin level

**Fig. 10:**
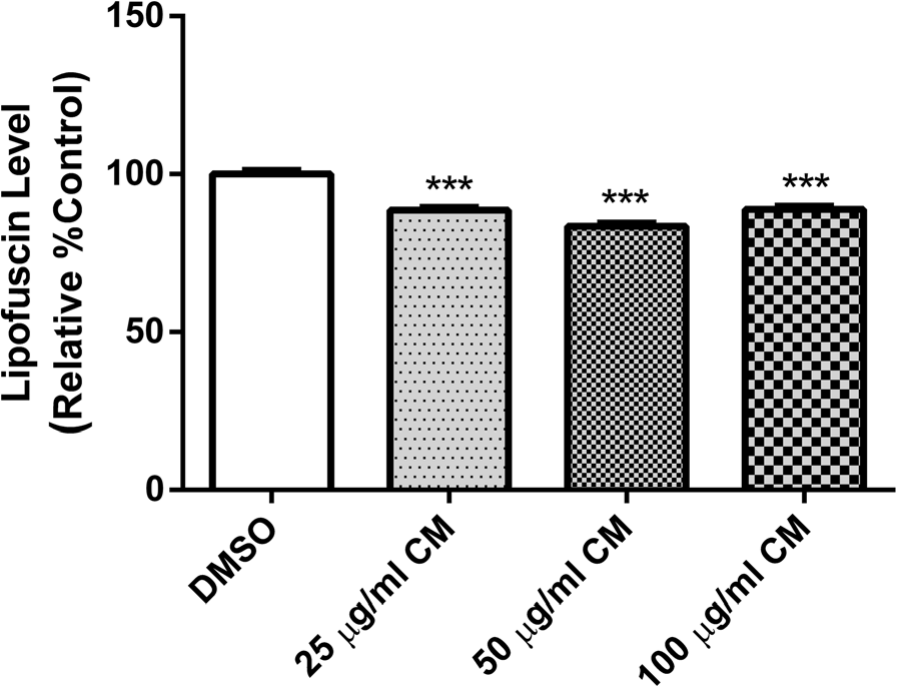
Effect of CM extract on aging pigment lipofuscin level. BA17 worms were treated with DMSO and CM extracts 25, 50 and 100 μg/ml. CM-treated worms significantly attenuate lipofuscin level. Values are mean ± SEM of at least 3 independent experiments. ***Different of DMSO control (*p* < 0.001)

Intestinal cells of *C. elegans* contain lysosomes and gut granules called lipofuscin, which are autofluorescent. Lipofuscin accumulation increases during oxidative stress and aging. The expression of lipofuscin was reduced in the BA17 transgenic worms treated with methanol extract (25, 50 and 100 μg/ml). The extract treatment at a concentration of 50 μg/ml showed the highest reduction of autofluorescence (16.60 ± 1.10%) when compared to the DMSO control group (*p* < 0.001) (Fig. 10).

### Effect of CM extract on lifespan extension

**Fig. 11:**
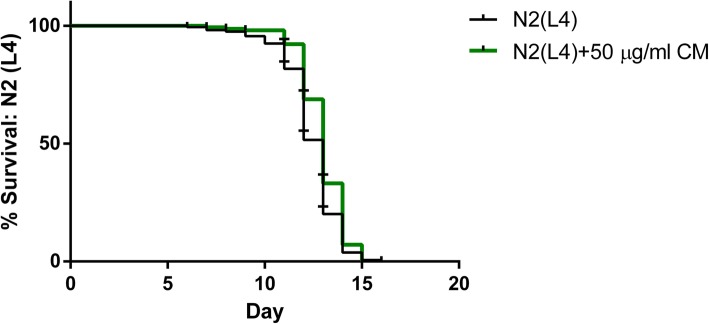
Longevity of N2 worm-treated with CM extracts. Lifespan extension after 50 μg/ml of CM extract treatment showed in cumulative survival plots

To evaluate anti-aging properties, we investigated the effect of CM extract on the lifespan of wildtype nematodes under normal conditions. The results showed that the extract at a concentration of 50 μg/mL was capable of enhancing the survival of wildtype N2 worms when compared to the DMSO control. The mean lifespan of the 50 μg/ml extract-treated L4-stage worms was 12.95 d, which was slightly longer than that of the control group, with a significant difference at *p <* 0.001 (Fig. 11 and Table 4).

**Table 4:** Results and statistical analyses of *C. elegans* lifespan assay

Treatment: N2 (L4)	Mean lifespan (day) ± SEM	% increased lifespan (vs. control)	*p-value* (vs. control)	*p-value* summary	Number of worms
Control	12.41 ± 0.12				*N* = 159
50 μg/ml CM extract	12.95 ± 0.10	4.35	0.0003	***	*N* = 154

The life span assay was carried out with wild type (N2) worms at 25 °C. Mean lifespan in days is the average number of days the worms survived in each group. The treatment group was compared to the control by log-rank (Mantel − Cox) tests followed by the Gehan − Breslow − Wilcoxon test

### Effect of CM extract on body length and brood size

**Fig. 12:**
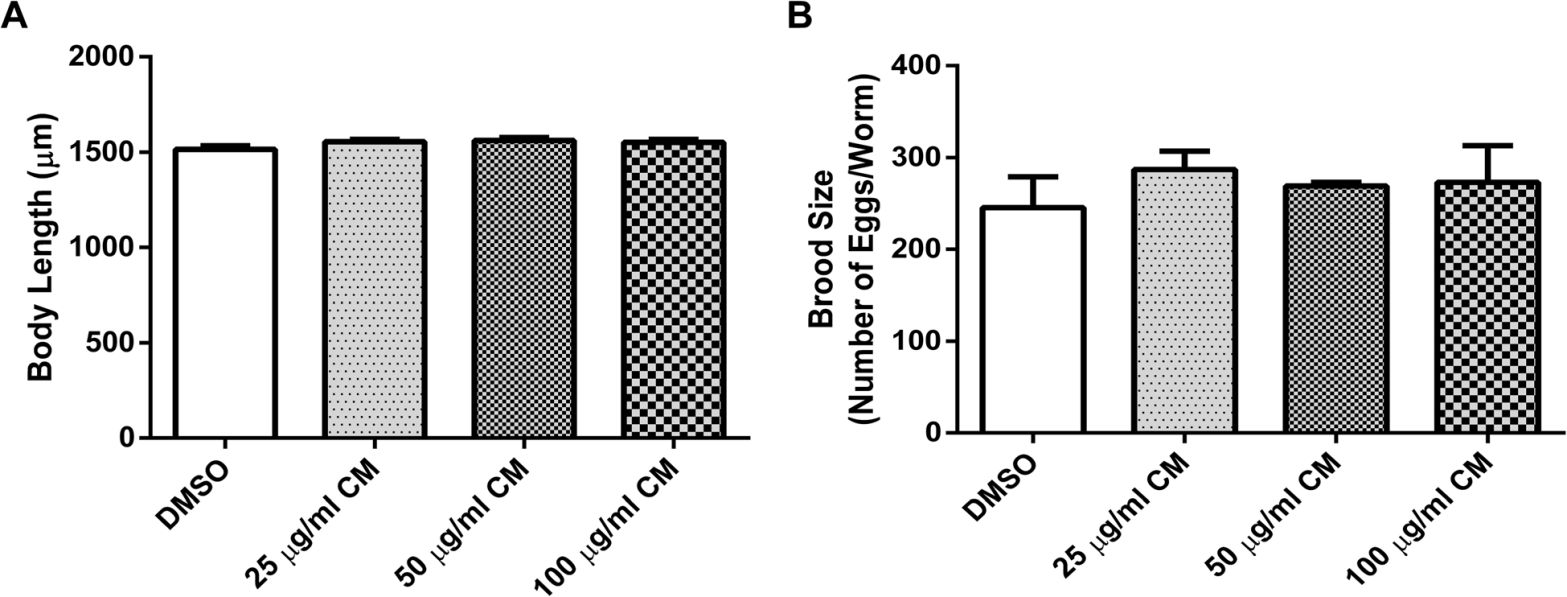
Effect of CM extracts on body length (**a**) and brood size (**b**) in N2 worms. Values are mean ± SEM of at least 3 independent experiments

To investigate the toxicity of the methanol extract, body length and brood size assays were performed to examine the development and fertility rate of the worms, respectively. These analyses could also be indicative of dietary restriction, which could also influence longevity. Analyses of body length revealed no difference in mean body length in worms treated with 25, 50 and 100 μg/ml extract when compared with the DMSO control (Fig. 12a). Additionally, the brood size assay did not show alterations in the number of eggs laid after extract treatment (Fig. 12b). Thus, dietary restriction can be ruled out.

## Discussion

Our study confirms previous reports that the methanol extract of *C. mimosoides* (CM) is rich in phenolics and flavonoids. The methanol extract exhibited the highest free radical scavenging capacity in vitro via DPPH and ABTS assays compared with hexane and ethyl acetate extracts. Similarly, previous studies of CM have obtained bioactive compounds from the polar substances rather than the nonpolar substances [8–11, 30, 31]. The major phenolic compounds of the extract identified by LC-MS in our study include gallic acid, theogallin, bergenin, 3-O-methylgallate, quercetin, clausarinol and emmotin A. This finding is similar to previous reports that gallic acid is a major constituent of the compounds in CM extract [10, 11, 31].

To further investigate a potential antioxidant activity of the methanol extract in vivo, *C. elegans* was employed as a model organism. The extract increased survival rates in *C. elegans* under juglone-induced oxidative stress and lowered intracellular ROS levels. In addition, HSP-16.2 was also significantly lower in CM-treated worms than in the control. Taken together, these results supported that the phenolics of CM are effective antioxidants in vivo and that they are bioavailable. These findings agree with several other studies that clearly showed an antioxidant effect of polyphenols [17–19, 32–34].

To investigate the mechanism underlying the antioxidant effect of CM extract, DAF-16/FOXO and SKN-1/NRF-2 pathways were monitored in this study. Normally, the transcription factor DAF-16 is localized in the cytosol in its inactive phosphorylated form. Oxidative damage can induce its activation by dephosphorylation and subsequent translocation into the nucleus. DAF-16 activation is responsible for stress response and lifespan extension [35]. Upon nuclear localization, DAF-16 induces the transcription of several genes involving antioxidant systems, such as SOD-3, which encodes mitochondrial superoxide dismutase (Mn-SOD). Previous studies suggested that this enzyme could protect the worms against ROS via elimination of free radicals [29]. The transcription factor SKN-1 regulates the expression of downstream genes of phase II detoxification enzymes. [36] GST-4 is an isoform of glutathione S-transferases that plays a major role in phase II detoxification process *in C. elegans* and can be activated by SKN-1. [37]

In the present study, we observed enhanced translocation of DAF-16 after treatment, whereas the localization of SKN-1 was not affected. The expression of SOD-3 and GST-4 genes were altered accordingly. Therefore, these results suggest that the extract exerts its antioxidant effect through the activation of DAF-16/FOXO pathway. Moreover, our findings are consistent with previous observations with polyphenol-rich plant extracts and isolated compounds that protect *C. elegans* against oxidative stress via the DAF-16/FOXO pathway [2, 19, 38–42].

Elevated ROS production apparently is a major contributing factor in the aging process [43]. Lipofuscin, an indicator of both oxidative stress and aging in *C. elegans* [44], is an autofluorescent pigment that accumulates progressively over time, particularly in lysosomes and gut granules of intestinal [45]. We found a lower accumulation of lipofuscin after CM treatment, which is in agreement with the observed antioxidant and anti-aging capacities of the extract. [18, 33, 34, 39, 46] Lifespan was slightly enhanced by the extract; this effect was not due to dietary restriction because neither the development nor the fertility of the worms were impaired.

## Conclusions

In conclusion, the current study demonstrates the relevant antioxidant and anti-aging activities of the CM extract in *C. elegans.* The extract was able to increase stress resistance and to reduce intracellular ROS levels as well as the expression of the HSP stress gene after exposure to oxidative stress. The extract was also able to enhance the nuclear localization of the DAF-16 transcription factor and the expression of the SOD-3 gene, demonstrating that its antioxidant activity is probably mediated through the DAF16/FOXO pathway. Overall, these results suggest that *C. mimosoides* could be a potential dietary supplement and alternative medicine with antioxidant and anti-aging properties. However, intervention studies with other organisms are required to corroborate our findings in *C. elegans*.

## Additional file

Additional file 1: Antimicrobial activity test using agar diffusion method. CM extract at the concentration 200 μg/ml showed no effect on *E. coli* OP50 (PDF 68 kb)

## Data Availability

All data generated or analysed during this study are included in this published article. The datasets used and/or analysed during the current study available from the corresponding author on reasonable request.

## Abbreviations

ABTS: 2,2-Azino-bis (3-ethylbenzothiazoline-6-sulfonic acid)
CM: *Caesalpinia mimosoides* L
DAF-16/FOXO: Forkhead box protein O
DMSO: Dimethyl sulfoxide
DPPH: Diammonium salt, 2,2-Diphenyl-1-picrylhydrazyl
EGCG: Epigallocatechin gallate
GAE: Gallic acid equivalents
GFP: Green fluorescence protein
GST-4: Glutathione S-transferase 4
H2DCF-DA: 2,7-dichlorofluorescein diacetate
HSP-16.2: Heat shock protein-16.2
Juglone: (5-Hydroxy-1,4-naphthoquinone)
LC-MS: Liquid chromatography-mass spectrometry
NaOCl: Sodium hypochlorite
NaOH: Sodium hydroxide
QE: Quercetin equivalents
ROS: Reactive oxygen species
SKN-1/Nrf-2: Nuclear factor erythroid 2-related factor 2
SOD-3: Superoxide dismutase-3
