# Supplementary material for: Leaf extract of Caesalpinia mimosoides enhances oxidative stress resistance and prolongs lifespan in Caenorhabditis elegans
Source: BMC Complement Altern Med. 2019 Jul 8;19:164. doi: 10.1186/s12906-019-2578-5 (PMC6615182; doi:10.1186/s12906-019-2578-5)
Supplement: Supplementary file 1 — Antimicrobial activity test using agar diffusion method. CM extract at the concentration 200 μg/ml showed no effect on E. coli OP50 (PDF 68 kb) [file 12906_2019_2578_MOESM1_ESM.pdf]

Additional File 1

Antimicrobial activity test using agar diffusion method

CM extract was proceeded the agar diffusion test to assess the sensibility of *E. coli* OP50. The bacteria was diluted in 0.9% NaCl broth to match 0.5 in the MaFarland densitometer. The bacteria broth was then spread onto LB agar plate. After allowed to dry, sterile pasteur pipette was used to make the wells by carefully stab into the agar. The wells were filled with 60 µl of CM extract (200µg/ml), 128 µg/ml ampicillin or DMSO. The plates were incubated at 37°C for 24h.

The result found that CM extract as well as DMSO had no effect on *E. coli* OP50. While the bacteria did not allow to grow in the ampicillin well. The result may suggest that CM extracts did not interfere with E coli in the experiments. Thus, the life extension and stress resistant effects of CM extracts may not depend on the antimicrobial activity, and the extracts may promote these effects by acting directly to the worms.

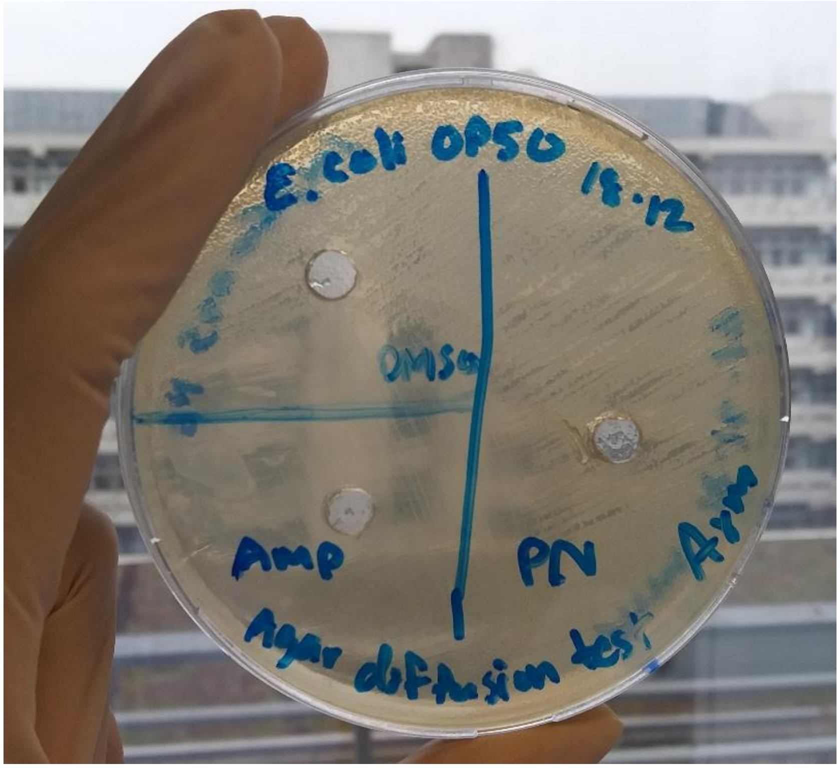

Antimicrobial activity test of CM extract against *E. coli* OP50. CM (PN was referred to Pak Nam, its Thai name) extract at the concentration 200 µg/ml. Ampicillin and DMSO were used as positive and negative control, respectively. CM extract showed no effect on *E. coli* OP50.
